# Residual limb volume fluctuations in transfemoral amputees

**DOI:** 10.1038/s41598-021-91647-9

**Published:** 2021-06-10

**Authors:** Linda Paternò, Michele Ibrahimi, Elisa Rosini, Giuseppe Menfi, Vito Monaco, Emanuele Gruppioni, Leonardo Ricotti, Arianna Menciassi

**Affiliations:** 1 The BioRobotics Institute, Scuola Superiore Sant’Anna, Pisa, Italy; 2 Department of Excellence in Robotics & AI, Scuola Superiore Sant’Anna, Pisa, Italy; 3 IRCCS Fondazione Don Carlo Gnocchi, 20148 Milan, Italy; 4 INAIL Centro Protesi, Bologna, Italy

**Keywords:** Rehabilitation, Quality of life

## Abstract

This study constitutes the first attempt to systematically quantify residual limb volume fluctuations in transfemoral amputees. The study was carried out on 24 amputees to investigate variations due to prosthesis doffing, physical activity, and testing time. A proper experimental set-up was designed, including a 3D optical scanner to improve precision and acceptability by amputees. The first test session aimed at measuring residual limb volume at 7 time-points, with 10 min intervals, after prosthesis doffing. This allowed for evaluating the time required for volume stabilization after prosthesis removal, for each amputee. In subsequent sessions, 16 residual limb scans in a day for each amputee were captured to evaluate volume fluctuations due to prosthesis removal and physical activity, in two times per day (morning and afternoon). These measurements were repeated in three different days, a week apart from each other, for a total of 48 scans for each amputee. Volume fluctuations over time after prosthesis doffing showed a two-term decay exponential trend (R^2^ = 0.97), with the highest variation in the initial 10 min and an average stabilization time of 30 min. A statistically significant increase in residual limb volume following both prosthesis removal and physical activity was verified. No differences were observed between measures collected in the morning and in the afternoon.

Clinical Trials.gov ID: NCT04709367.

## Introduction

Despite the advancements in the prosthetic design and the enhancements concerning wearable robotic platforms^1,2^, most amputees still complain about discomforts related to the prosthetic physical human–machine interface (pHMI), i.e. the socket^3–6^. An optimal prosthetic socket must be comfortable for the user, while ensuring stable fitting and proper load transmission, especially in lower limb prostheses^7^. These requirements are conditioned by residual limb volume fluctuations and changes, herein defined as volume variations occurring during the days and over months/ years, respectively. In fact, residual limb volume fluctuations and changes can compromise the prosthesis fitting which can, in turn, cause relative socket-residual limb movements, alter the stress distribution on tissues, involve dermatological problems (e.g., ulcers, irritations, vascular occlusions, dermatitis, blisters) and pain for the user^3,8,9^.

Residual limb volume changes, especially reduction, are particularly relevant during acute and post-acute operative recovery periods (i.e., 12–18 months after amputation), because of the oedema reabsorption and the muscle atrophy following the surgical procedure^10–13^. Regardless of the post-operative phase, volume changes occur in stabilized amputees (i.e., > 18 months since amputation) as well. In this regard, both increases and decreases in residual limb volume have been reported and volume gains are generally due to body weight increase^11–13^, mainly associated to a common impairment of the subject’s activity level.

Peripheral vascular disorders (e.g., increased blood pooling in venous compartment, excessive arterial vasodilatation and changes in interstitial fluid volume) can affect bodily fluid movements in the residual limb, thus causing mostly volume fluctuations^12,14^. Physical activity, prosthesis suspension system and socket size can further exacerbate these phenomena. As matter of facts, earlier studies have documented an increased volume after prosthesis doffing when vacuum suspension systems are used; noticeably, the rate of variation depends on the applied vacuum pressure^12,15–17^. On the contrary, suspensions not based on vacuum, e.g., pin locking systems, seem to mainly cause volume reductions after prosthesis removal^18^. Moreover, diet, weather conditions, comorbidities, and several other factors can impact these variations both in the short and long terms^14^. All in all, these factors involve a rate of variation in volume ranging from − 11 to + 11%^12,14,19,20^ in the short term and from − 4.5 to + 21%^11,12,21^ in the long term, concerning stabilized transtibial amputees. As a result, the fitting of the “definitive” prosthesis is continuously jeopardized and the socket shape must be frequently adjusted by prosthetist.

Despite the widely documented variation of residual limb volume in transtibial amputees, to the best of our knowledge, no reliable data pertaining to transfemoral amputees are available in literature (see Table S1 in supplementary material). The skewed distribution of studies toward the population of below-knee amputees can be ascribed to different reasons such as the more straightforward measurement set-up and the more compelling need to reduce pain in bony regions. Specifically, an improper fitting of the prosthetic socket may involve high stresses on soft tissues more frequently in transtibial amputees than in transfemoral ones, because of the wide bony prominences at the residual limb-socket interface^3^. On the other hand, the larger volume of soft tissues in transfemoral residual limbs can be subjected to even larger fluctuations^22^, highly affecting comfort and fitting of the prostheses. Accordingly, volume fluctuations in the residual limb of transfemoral amputees deserve to be analyzed in depth to provide suitable reference values for the design of novel smart adjustable sockets, similarly to what has been done for transtibial prostheses^23^.

This work aims at filling this gap in the state-of-the-art and to quantify the volume fluctuations during the day in the stabilized transfemoral amputee population. To achieve this goal, volume fluctuations due to prosthesis doffing and physical activity had to be investigated on a statistically significant number of subjects, both in the morning and in the afternoon. Moreover, to improve the data reliability, the protocol had to be repeated at least three times in three different days for each amputee.

## Measurement systems for the assessment of residual limb volume

Residual limb volumes can be measured through many techniques, as widely described by Sanders et al.^12^. In this section, we will briefly recapitulate them in order to clarify the rationale undergoing the methodological approach used in this study.

The simplest measurement system for the assessment of residual limb volume consists in dipping the residual limb or its cast within a box filled with water, and measure the water displacement^20^. However, this technique is susceptible to errors due to subject’s movements and surface tension at the limb-water interface, thus resulting in a low reliability^12^.

Anthropometric models can be reconstructed by importing anatomical landmarks distances, measured by tapes or calipers, but these models are not accurate enough to guarantee reliable results^24,25^. Furthermore, as all techniques involving contact with tissues, anthropometric measurements influence the residual limb shape during the evaluation^12^.

Magnetic resonance imaging, ultrasound and spiral X-ray computed tomography can detect variations in volume and internal residual limb structures. Nevertheless, they are costly, invasive, affected by errors due to subject’s movements, and require extensive post-processing. In addition, they are time-consuming and not fast enough to allow for measurements of volume fluctuations due to prosthesis doffing.

More recently, Sanders et al.^26–30^ developed a bioimpedance device to measure the conductive tissue extracellular fluid (ECF) volume of transtibial residual limbs while donning the prosthesis. Through this approach, the assessed ECF volume fluctuations mainly refer to muscles and skin, without including bone and adipose tissues. Accordingly, this approach can only document relative variations of the residual limb volume. In this regards, outcomes cannot be directly comparable to those resulting from state-of-the-art measurement devices dealing with variation of the absolute volume.

Measurement strategies comprising the use of a portable 3D scanner are among the most efficient solutions, as demonstrated by de Boer-Wilzing et al.^31^. Thanks to the recent developments in 3D scanning, these systems are nowadays reliable, safe, fast and portable. All these features are fundamental for clinical applications^14,32,33^. Dickinson et al.^34^ have evaluated the accuracy of three hand-held 3D scanners: high reliability and accuracy for the VIUScan marker-assisted laser scanner and the Go!SCAN 3D optical scanner were demonstrated (both metrology-grade scanners of Creaform Inc; Canada). Moreover, the Go!SCAN50 scanner allows for a specific body-scanning option (namely semi-rigid positioning), consisting of an algorithm implementation within the acquisition software able to compensate small body tremors associated to the hand holding the scanner and to the scanned object. Furthermore, marker dots are not needed to be applied on the object to be scanned, thanks to the system ability to capture the object natural features. Thus, a 3D scanner based approach including the Go!SCAN50 was selected for the assessment of the volume fluctuations of transfemoral residual limbs in the framework of this study.

## Methods

### Subjects

This study was approved by the ethical committee “Area Vasta Emilia Centro, Regione Emilia-Romagna CE-AVEC” (protocol ID: P-PPRAI1/2-01, CE protocol reference number: 105/2018/OSS/AUSLBO, date of registration: 11/05/2018; ClinicalTrials.gov ID: NCT04709367, date of registration: 12/01/2021) and carried out at the INAIL Prosthetic Center (Bologna, Italy). All experiments were undertaken in accordance with the World Medical Association’s Code of Ethics and the Declaration of Helsinki. All recruited subjects signed an informed consent before starting experimental sessions.

The inclusion criteria determined the involvement of stabilized (i.e., time since amputation > 18 months) transfemoral amputees between 18 and 65 years old. Subjects with concurrent medical issues or unable to safely perform the physical tasks required in the experimental protocol were excluded.

According to the literature^35^, in order to identify a suitable sample size, we focused our attention on the effects of the physical activity on the volume fluctuation, since it was expected to be one of the main factors involving larger volume variations. Hence, to identify the target number of subjects needed to obtain a statistical power of 95%, a preliminary study was carried out on 6 transfemoral amputees, to measure residual limb volume fluctuations due to physical activity. Results of this preliminary study are reported in our previous work^36^. Then, using these data, the following equation was applied for the sample size estimation (paired t test)^35^:

1 $${\mathrm{n}=\left[\frac{\left({\mathrm{z}}_{\mathrm{\alpha }}+{\mathrm{z}}_{\upbeta }\right)\upsigma }{\updelta }\right]}^{2}$$

where β is the type II error probability (0.05) for the desired statistical power of 95% (power = 1 − β), α the desired significance level (0.05), *z*_α_ and *z*_β_ the standard normal scores for confidence level α and β respectively, σ the population standard deviation (0.051 dm^3^), and δ the expected difference (0.040 dm^3^). Particularly, σ and δ were evaluated by using preliminary data^36^. Indeed, they can be approximated as the mean and the standard deviation of the difference between residual limb volumes measured after and before physical activity. Thus, the target subjects number, $\mathrm{n}$, resulted equal to 24.

### Experimental setup and data acquisition

**Figure 1:**
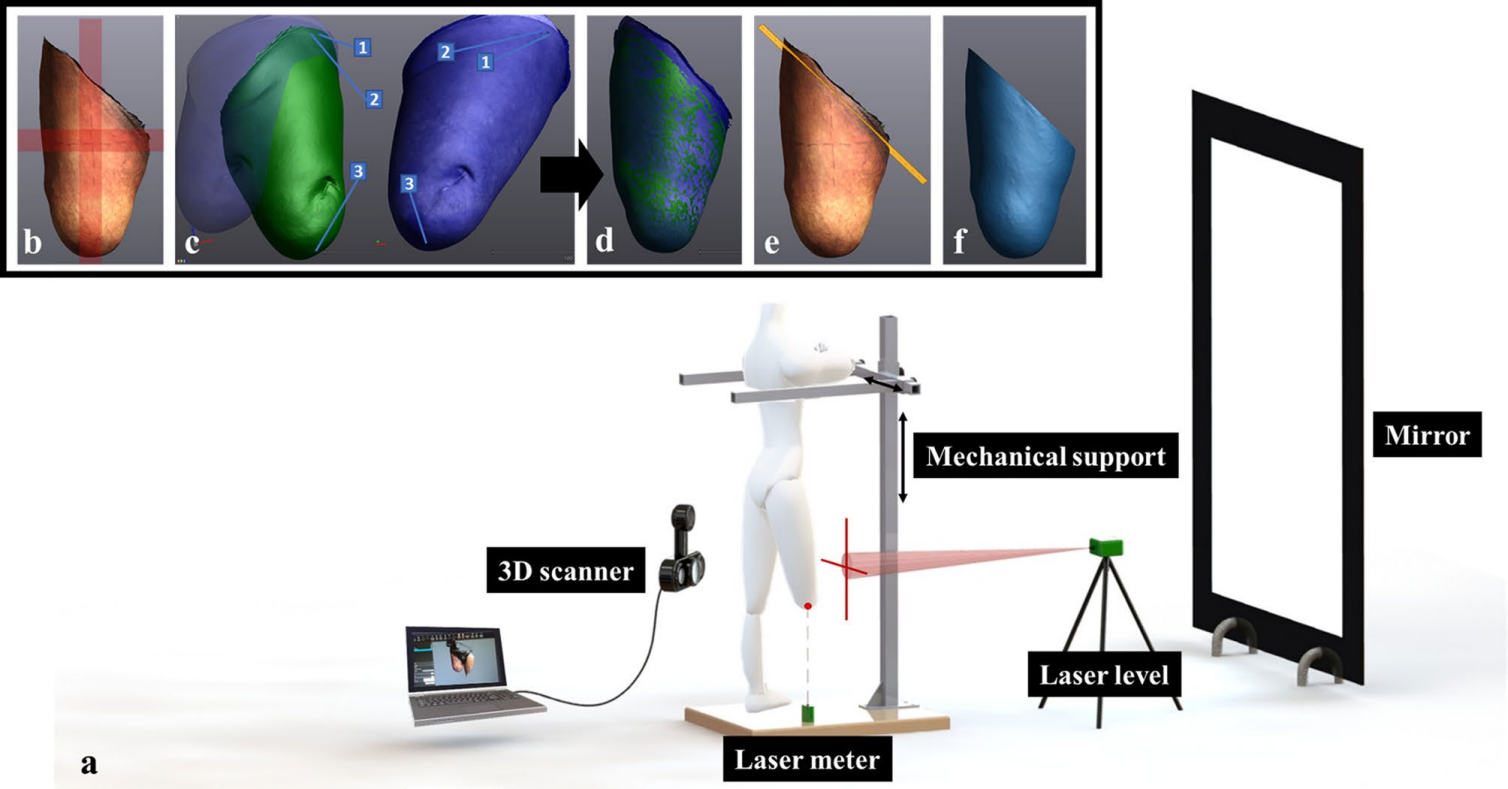
(**a**) Experimental set up for transfemoral residual limb 3D scanning; (**b**) residual limb 3D scan (in red a schematic representation of the two perpendicular lines projected by the laser level on the anterior part of the residual limb); (**c**) the pre-align option of the Surface Best-Fit alignment method: fixed model in blue and mobile model in green; (**d**) alignment result; (**e**) scan cutting plane in orange; (**f**) final mesh. These images were created using VXelements 6.0 (www.creaform3d.com) and SOLIDWORKS 2020 software (www.solidworld.com).

To yield the protocol reliable and acceptable for the enrolled amputees, a dedicated experimental set-up was developed (Fig. 1a). It included a mechanical support, adequately designed to help the enrolled amputees standing on the sound limb in a stable and comfortable way during scanning. For each subject, a paper sheet was glued on the base of the mechanical support to draw the footprint in the initial standing position. A laser level and a laser meter were used to project two perpendicular lines on the anterior surface of the residual limb and a dot on the distal end, respectively. Such tools and the mechanical support were positioned at the beginning of the protocol for each amputee and were kept in position until the end. The two lines and the dot, projected on the residual limb, as well as the footprint, were drawn before starting the tests, to identify the same limb orientation for all the scans, thus allowing the subjects to sit in a chair positioned behind them at the end of each scan. A mirror was placed in front of the amputee to allow for visual feedback, hence helping to find and maintain the same position during scanning.

Before starting, four dots were drawn on the residual limb as anatomical landmarks to uniquely identify a scan cutting plane; they were used afterwards in the post-processing of the 3D image data. One dot was drawn on the ischial tuberosity, one on the external surface of the greater trochanter, and the two remaining dots were drawn on a horizontal axis, about 1 cm distally with respect to the great trochanter, and located about 5 cm anteriorly and 5 cm posteriorly on the skin (see Video 1 in supplementary material). All dots were drawn by an expert prosthetist which identified the bony prominences by palpation. These body regions were selected since usually featured by minimal volume fluctuations because of the presence of bony structures with a few soft tissues^12^.

Scan files were acquired with the VXelements software (Creaform Inc; Canada), that allows for real-time visualization of the 3D image data (Fig. 1b, see Video 1 in supplementary material). Once the acquisition was completed, the mesh optimization was carried out (i.e., filling holes, eliminating bad frames, performing data clean-up, smart decimation). Then, the meshes were imported in the VXmodel software (Creaform Inc; Canada) for post-processing. Three different options can be chosen in the software for aligning scans: (1) Global Registration, (2) Surface Best-Fit alignment and (3) N-Point alignment. To select the best tool, 3 consecutive scans of the thigh were performed on 4 not-amputated subjects standing on one leg on a step positioned at the base of the mechanical support, thus resulting in a total of 12 scans. Based on these data, the Surface Best-Fit alignment was selected (Table 1) since it involves the smallest volumetric error, as averaged across subjects.

**Table 1:** Mean ± standard deviation of the volumetric error [%] of the 3 consecutive scans of a lower limb of 4 not-amputated subjects, for the three alignment tools of the VXmodel software.

Global registration	Surface best-fit	N-Point
0.338 ± 0.097	0.313 ± 0.072	0.315 ± 0.245

The Surface Best-Fit tool aligns the meshes using their common surface when they are not in the same referential by considering one mesh fixed. Thanks to the Pre-align option of the tool, it was possible to select at least 3 points on the fixed mesh, and then the same points on the mobile one (Fig. 1c). Thus, the dots drawn on the residual limb as anatomical landmarks—visible in the acquired scan textures—were used (see Video 1 in supplementary material). Once the common points were selected, the Surface Best-Fit alignment was completed (Fig. 1d). Since the software allows for cutting meshes along planes, the point drawn on the ischial tuberosity, and the other two about 1 cm distally with respect to the great trochanter, were used to define the scan cutting planes (Fig. 1e). The resulted holes were filled in a planar way and the volume was computed by the software (Fig. 1f).

### Experimental protocol

Results reported in reported in our previous work^36^ highlighted that each amputee may require a different period of time to reach a volume stabilization after doffing the prosthesis. Hence, the experimental protocol was constituted of four test days and defined as follows.

**Figure 2:**
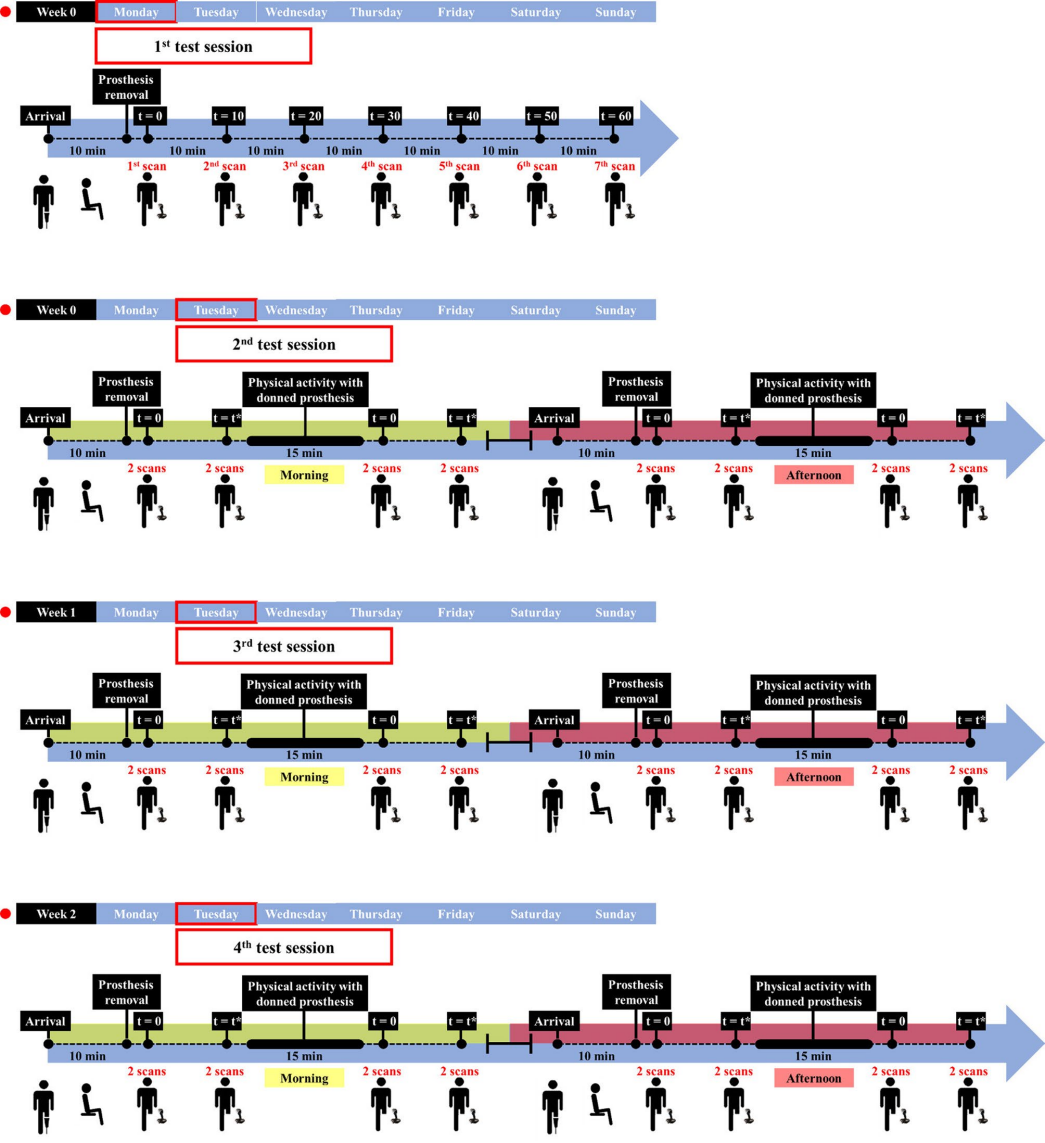
Experimental protocol for 1st test session (up) and for the other three ones (bottom). At the end of each scan, the subjects sat in a chair positioned behind them and remained sitting until the following scan. t*: stabilization time evaluated during 1st session for each amputee. This image was created using PowerPoint (www.microsoft.com).

1st session: during this test session (Fig. 2—Monday week 0), a resting period of 10 min was scheduled upon arriving in order to reach a homeostatic condition of the limb within the prosthesis. Then, the prosthesis was doffed, the amputee was helped to reach the mechanical support of the experimental set-up and 7 scans were acquired at intervals of 10 min in a standing position. This session allowed for the characterization, over time, of the residual limb volume fluctuations due to prosthesis removal and for the identification of the time required to stabilize the residual limb volume for each amputee. More in detail, volume change was calculated starting from minute 20 and until the change between successive time points was lower than the error evaluated for the 3D body scanning method (i.e., 0.313%; TABLE 1).By that time, volume was considered stabilized.

2nd session, 3rd session, 4th session: further three sessions of tests were performed in three different days, a week apart from each other (Fig. 2 – Tuesday week 0, Tuesday week 1, Tuesday week 2). Each session was featured by two testing times, one in the morning and one in the afternoon. During both (morning and afternoon), upon arrival, the amputee rested for 10 min with the prosthesis donned. Then, 2 consecutive scans were performed immediately after prosthesis doffing. Other 2 consecutive scans were carried out after the amputee’s stabilization time (evaluated in the 1st session). Then, the amputee donned the prosthesis and 15 min of physical activity were performed (i.e., walking at a self-selected speed on a treadmill) and the same scanning sequence (i.e., 2 scans just after doffing the prosthesis and 2 scans after the residual limb volume stabilization) was repeated. This resulted in 48 scans for each amputee.

### Statistical analyses

All statistical tests were carried out in IBM SPSS Statistics environment and the significance level was set equal to 0.05.

1st session: the normality of the volume data acquired in this test session was verified (Kolmogorov–Smirnov’s test and Shapiro–Wilk’s test), while the assumption of sphericity was violated (Mauchly's test) ($\mathrm{p }= 0.05$). Accordingly, the one-way ANOVA with repeated measures and the Greenhouse–Geisser correctional adjustment was used to investigate the effects of the factor time (7 levels; i.e., time points at 10 min interval) on the measured volume (H_0_: no difference among sample means at different time-points). Then, Bonferroni post-hoc comparisons were carried out.

The mean and the standard deviation of the post-doffing volume fluctuations over time were calculated, using the first scan ($\mathrm{t}=0$, Fig. 2—Monday week 0) as the reference. Then, the curve trend of the measured data was fitted in Matlab R2018a. As found in literature for transtibial amputees^14,29,37,38^, the following two-term exponential decay function was used to curve-fit mean volume fluctuations versus time:

2 $${\Delta \mathrm{V}}_{\mathrm{t}}={\Delta \mathrm{V}}_{1}\left(1-{\mathrm{e}}^{-\mathrm{kt}}\right)$$

2nd session, 3rd session, 4th session: during each session, volumes were computed and averaged between the 2 consecutive scans resulting at each time-point (Fig. 2). This resulted in 8 volume values per day for each amputee. Afterward, these volume values were averaged over the three different test days, resulting in 8 values for each amputee at the specific time-points of the day. The normality (Kolmogorov–Smirnov’s test and Shapiro–Wilk’s test) and the sphericity (Mauchly's test) of data distribution were preliminarily verified. Then, the three-way ANOVA with repeated measures was performed to investigate the effects of factors: testing time (two levels: morning vs afternoon), physical activity (two levels: before vs after physical activity), prosthesis removal (two levels: immediately after prosthesis doffing vs after the stabilization time), and their interactions on measured volume.

### Consent for publication

Ethics approval was obtained from the ethical committee “Area Vasta Emilia Centro, Regione Emilia-Romagna CE-AVEC”. Protocol ID: P-PPRAI1/2-01. CE protocol reference number: 105/2018/OSS/AUSLBO.

## Results

### Subjects and baseline condition (1st session)

The general features of the enrolled amputees are summarized in Table 2. All subjects were traumatic amputees. Only one female took part in the study. The 20.8% of subjects reported a recent amputation (2–5 year) and the 79.2% was chronic (> 5 year). The majority of enrolled subjects wore a quadrilateral socket (54.2%) and a suction suspension system based on a unidirectional valve (91.7% in total: 50% without a liner and 41.7% with a Seal-In liner). The mean self-selected speed during walking on the treadmill resulted equal to 0.6 ± 0.1 m/s.

**Table 2:** Subjects’ general features, time required to stabilize the residual limb volume, t* [min], and self-selected speed on the treadmill, v [m/s].

	Age	t since amp	K	Socket Design	Socket Susp.	t*	v
S1	42	14	K3	CAT-CAM	Seal-In	40	0.4
S2	63	33	K2	ICS	Suction	20	0.4
S3	58	5	K4	Sub-Isc	Seal-In	30	0.6
S4	59	13	K3	ICS	Seal-In	40	0.6
S5	44	15	K4	ICS	Suction	30	0.7
S6	48	12	K3	MAS	Suction	40	0.8
S7	62	44	K4	Quad	Suction	40	0.5
S8	59	42	K3	Quad	Seal-In	30	0.6
S9	46	22	K4	Quad	Suction	30	0.5
S10	51	11	K3	Quad	Suction	20	0.9
S11	58	10	K4	Quad	Seal-In	30	0.7
S12	47	2	K3	Quad	Pin- liner	50	0.7
S13	46	2	K4	Quad	Suction	20	0.8
S14	42	14	K3	ICS	Seal-In	20	0.7
S15	41	5	K4	Quad	Seal-In	20	0.7
S16	56	9	K3	Quad	Seal-In	20	0.8
S17	60	5	K2	Sub-Isc	Seal-In	30	0.5
S18	39	13	K3	Sub-Isc	Seal-In	40	0.6
S19	57	36	K2	Quad	Suction	50	0.7
S20	65	49	K3	Quad	Suction	60	0.4
S21	65	56	K4	Quad	Suction	30	0.5
S22	62	45	K4	Quad	Suction	20	0.7
S23	49	24	K3	ICS	Mechanic	20	0.5
S24	60	23	K3	Quad	Suction	30	0.7
M ± std	53.3 ± 8.4	21.1 ± 16.2				31.7 ± 11.3	0.6 ± 0.1

*S* subject; age and t since amputation in years, *K* K level, rating system used to indicate the individual's potential functional ability, *K2* activities typical of limited community ambulatory, *K3* activities typical of community ambulatory, *K4* high-impact activities, *CAT-CAM* Contoured Adducted Trochanteric-Controlled Alignment Method, *ICS* Ischial Containment Socket, *Sub-Isc* Sub-Ischial, *MAS* Marlo Anatomical Socket, *Quad* Quadrilateral, *M* mean.

**Figure 3:**
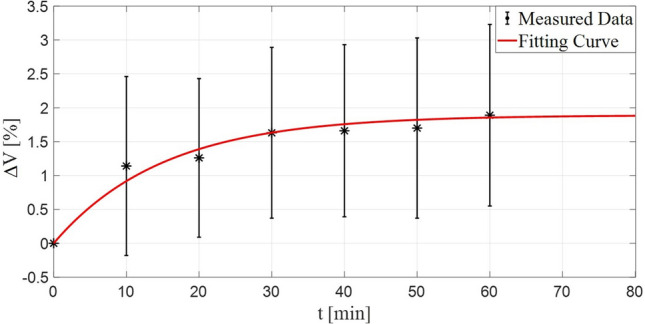
Post-doffing volume fluctuations plotted against time: mean and standard deviation in black, two-term exponential decay fitting curve in red.

Among the recruited subjects, 22 completed the 1st test session, while 2 only performed 5 out of 7 scans (Table S2 in supplementary material). Results revealed that prosthesis doffing produced an increase in residual limb volume, with the highest change rate in the first 10 min (Fig. 3). In particular, the amputees’ residual limbs required, on average, 30 min to stabilize in volume (see t* in Table 2; Fig. 3).

The repeated measures one-way ANOVA test with Bonferroni post-hoc comparisons confirmed these results, showing a significant differences for all time-points compared to $\mathrm{t}=0$ ($\mathrm{p }< 0.05$, Table 3).

**Table 3:** Results of repeated measures one-way ANOVA test with Bonferroni post-hoc comparisons.

Test of within-subject effects							
	Source	Type III sum of squares	df	Mean square	F	P	Partial eta square
Time	GG	0.040	3.438	0.012	9.828	**0.000**	0.319

Result of Bonferroni post hoc comparisons							
(I)	(J)	Mean (I–J)	Std. error	P	95% conf int diff		
					LB	UB	
t = 0	t = 10	− 0.031*	0.008	**0.023**	− 0.060	− 0.003	
	t = 20	− 0.032*	0.007	**0.002**	− 0.054	− 0.009	
	t = 30	− 0.040*	0.007	**0.000**	− 0.064	− 0.017	
	t = 40	− 0.047*	0.008	**0.000**	− 0.075	− 0.019	
	t = 50	− 0.044*	0.008	**0.001**	− 0.073	− 0.016	
	t = 60	− 0.053*	0.010	**0.001**	− 0.089	− 0.017	

*LB* lower bound, *UP* upper bound.

The two-term exponential decay function (Eq. 2) showed a good fit (R^2^ = 0.97) of mean volume fluctuations versus time, with ${\Delta \mathrm{V}}_{1}$ and $\mathrm{k}$ equal to 1.80% and 0.08 min^−1^, respectively.

Notably, the maximum measured volume change among all subjects was found equal to + 5.92%.

### Volume fluctuations within a day: 2nd session, 3rd session, 4th session

**Figure 4:**
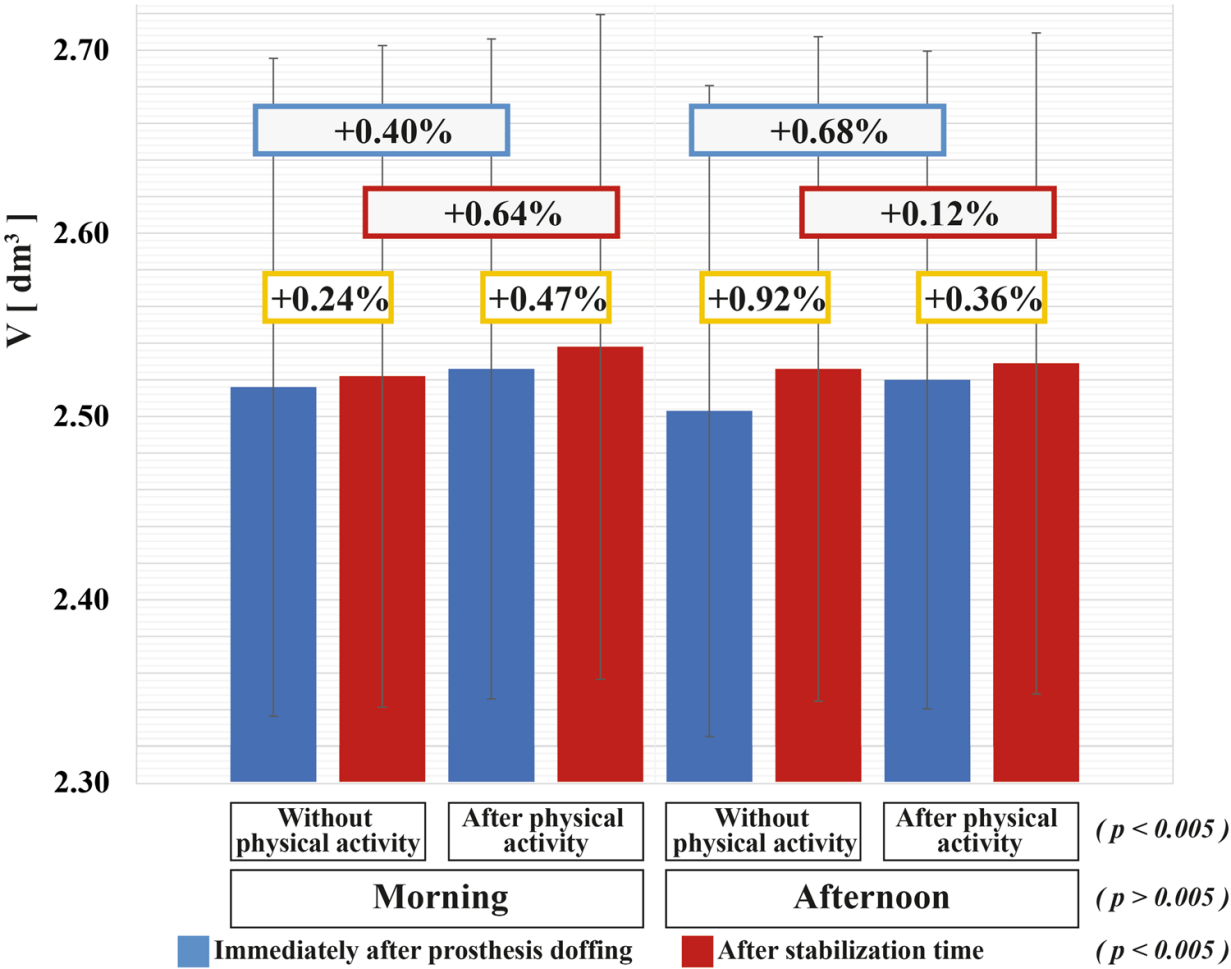
Mean and standard error of volume values at different times and significance of the three within-subject factors.

Among the 24 recruited amputees, 1 dropped out of the study after the 1st test day, resulting in 23 amputees. Results of the 3-way ANOVA with repeated measures showed no statistical differences for testing time ($\mathrm{p}> 0.05$), and a significant effect of both prosthesis removal $(\mathrm{p }< 0.005$) and physical activity ($\mathrm{p }< 0.005$) (Table 4; Fig. 4). Specifically, after removing the prosthesis, and after the physical activity, the residual limb volume increased, on average, of + 0.50% and + 0.46%, respectively.

**Table 4:** Results of repeated measures three-way ANOVA test.

Test of within-subject effects		
	F(2, 22)	P
Testing time (1)	1.195	0.286
Physical activity (2)	10.862	**0.003**
Prosthesis removal (3)	10.862	**0.003**
1 × 2	0.314	0.581
1 × 3	4.263	0.051
2 × 3	1.705	0.205
1 × 2 × 3	6.024	**0.022**

Within-subject factors: (1) testing time: morning or afternoon; (2) physical activity: before or after; (3) prosthesis removal: immediately after prosthesis removal or after stabilization time.

A significant effect was also observed for the interaction among all the three within-subjects factors (i.e., testing time × physical activity × prosthesis removal) ($\mathrm{p}< 0.05$, Table 4). Specifically, results revealed that the residual limb volume increased during the day, in particular after physical activity and prosthesis removal.

Notably, the maximum volume fluctuation due to the prosthesis removal as a percentage of the value immediately after the prosthesis doffing, across all subjects, ranged between − 4.18 and + 2.65% (mean $\pm$ std: + 0.35% $\pm$ 1.18%). Noticeably, the maximum volume reductions were always verified for subject 12, which is the only recruited amputee with a pin locking suspension system (Table 2). Also, the volume change due to the physical activity was evaluated for each subject as a percentage of the value before activity, resulting in a total change range equals to − 1.43% ÷  + 3.19% (mean $\pm$ std: + 0.51% $\pm$ 0.99%).

## Discussion

This study focused on the volume fluctuations affecting the residual limbs of transfemoral amputees. At first, the volume fluctuations after the prosthesis doffing was characterized over time. Then, the effect of the prosthesis removal and the physical activity was investigated within a day—comparing morning and afternoon results—and repeating the tests during three different sessions, 1 week apart from each other.

A specific experimental set up was realized, including a portable metrology-grade 3D scanner, namely the Go!SCAN50, that was identified as the most suitable solution for the study. A mechanical support for amputees in standing position was designed to increase the protocol acceptability and improve the measurements precision. The final volumetric error of the 3D body scanning method was found equal to 0.3%. This value ensures a high reliability of the obtained results if compared to other measurement approaches described in the literature. Indeed, the water displacement method showed a measurement error between 2.1 and 3.7% when directly applied on residual limbs^39^. The error is improved to $\approx$ 1% when residual limb casts are measured^40^ but they cannot perfectly replicate the residual limb volume. Anthropometric measurements resulted in an error between 2.4 and 5.7%^12^, while contact probes 3.7% on average^41–43^ and spiral X-ray computed tomography $\approx$ 1%^44,45^. Better results were reported when using custom optical scanners (0.6–0.8%)^46^ or laser scanners (0.5–0.4% on residual limb casts)^47,48^.

The study included 24 amputees, a number guaranteeing a statistical power of 95% with an α-value of 0.05 by using preliminary data^36^. However, only 22 subjects completed the required scans of the 1st test session, while 1 subject dropped out the subsequent ones. This resulted in a statistical power of 93% and 94%, respectively (α = 0.05).

The homogeneous features of the recruited population (Table 2) can be easily attributed to the recruiting prosthetic center, that is a national rehabilitation facility for work-related disabilities (INAIL, Italian National Institute for Insurance against Accidents at Work). This prosthetic center mostly deals with traumatic amputations due to work-related accidents; thus, it introduced a bias in the recruitment, as also described for other clinical studies^49^, and may likewise have contributed to the predominance of male amputees. Indeed, only one female subject was enrolled and all subjects reported a traumatic amputation. It has to be underlined that subjects with an amputation due to peripherical vascular diseases are expected to experience larger volume fluctuations^12^.

The protocol consisted of four test sessions in four different days for an overall duration of 3 weeks. During the 1st session, amputees’ residual limb volume was measured 7 times at intervals of 10 min after the prosthesis removal. As reported in literature on transtibial amputees^14,29,37,38^, a two terms exponential decay function demonstrated to curve-fit well the data (R^2^ = 0.97). Generally, residual limbs increased in volume after doffing the prosthesis (maximum measured value across all subjects equals to + 5.9%). The greatest volume change was found in the initial 10 min (Fig. 3). Then, values stabilized after 30 min on average (Table 2).When residual limbs are within the socket, interstitial pressure is expected to be higher because of the rigid socket constraint^12^. Then, socket doffing allows a release and reduction in terms of interstitial fluid pressure. Thus, an increment of the amount of fluid from arterial vessels into the interstitial space may occur, as well as a reduction from the interstitial space into the venous vessels^30^ As a consequence, the residual limb usually increases in volume when the prosthesis is removed. In addition, this effect may have been enhanced by the negative pressures applied on residual limb tissues by the prosthesis suspension system. Indeed, 22 among 24 enrolled amputees used a vacuum suspension (i.e., suction by unidirectional valve with or without a Seal-In liner, Table 2). Negative pressures on tissues mainly draw in body fluids, differently from the drawing out effect of positive pressures^29^. It is worth remarking that the interaction and the magnitude of these body mechanisms can be influenced by a complex interplay of factors: physiological and biometric parameters (e.g. blood pressure and BMI), age, lifestyle (e.g. smoking and activity level), residual limb features after amputation, socket shape and suspension system, etc. This accounts for the large variability among experimental results.

The increment in volume due to the prosthesis doffing was also confirmed by the results obtained in the following three test days. In particular, during these sessions, the residual limb volume was measured immediately after the prosthesis doffing and after the amputee’s stabilization time, before and after 15-min of walking on a treadmill, both in the morning and in the afternoon. The adopted stabilization time resulted from the 1st test day for each amputee. The data were analyzed by a repeated measured three-way ANOVA test (Table 4). Both prosthesis removal and physical activity caused a significant increase in the residual limb volume (Fig. 4). Generally, physical activity generates a blood pressure increment and an enhanced blood circulation, influencing the body fluids distribution. This can have led to the volume gain of the residual limb. In addition, the volume increment could be due to the pressure distributions applied on the residual limb tissues by the prosthesis socket and suspension system. Indeed, it is known that cyclic changes of pressures at the prosthetic pHMI continuously occur during walking—negative pressures in the swing phase and positive pressures in the stance phase^20^.

Furthermore, the application of vacuum due to the suspension system causes an increment of the negative pressures during the swing phase and a reduction of the positive pressures during the stance phase^8^. This influences the blood circulation and the drawing in/out body fluids, suggesting an increment in volume.

In addition, volume fluctuations within a day, albeit not statistically significant, were also observed, resulting in an overall range across subjects of − 4.2 to + 2.6% due to the prosthesis removal and − 1.4 to + 3.2% due to the physical activity. However, it needs to be stated that high volume reductions were verified only in subject 12, which used a pin locking suspension system for the prosthesis. Then, the positive pressures applied on the residual limb tissues might have caused a drawing out effect of the body fluids, when doffing the prosthesis. Moreover, these great volume change ranges might have been impacted by several other factors, depending on the specific subject and test day, e.g., diet, weather condition, comorbidities, hydration, medication etc. This complex interplay of factors can make difficult to derive general conclusions.

These data point out the critical need for an optimal pHMI interface for transfemoral prostheses, able to adapt comfortably and effectively to the residual limb. Indeed, these volume variations are enough to generate severe discomforts for amputees and, in the worst cases, impediments in donning the prosthesis^17^. Indeed, previous studies report difficulties to wear the prosthesis if a volume increase of + 3 to + 5% was verified^12,50^. In this regard, manually adjustable sockets can be used to re-adapt the socket volume and shape by users. However, a high risk of excessive tightening and improper stress distribution exists. To avoid severe consequences in the long term, e.g. residual limb deformation and mass loss, devices able to automatically adjust their volume and set correct stresses at the interface are needed.

On the other hand, the residual limb volume reductions can compromise the prosthesis fitting and cause altered stress distributions. As a consequence, excessive distal tip loading and high shear stresses can occur, thus causing pain, tissue injuries and other dermatological problems. In addition, a large residual limb volume loss can generate relative movements between the limb and the socket, thus affecting the prosthesis stability and increasing the risks of falling^51,52^. To achieve the challenging objective of an optimal prosthetic interface, the socket system should be able to compensate for these fluctuations in volume.

Overall, the results reported in this study advance the state-of-the-art concerning the volumetric fluctuations of transfemoral residual limbs. Furthermore, they provide the requirements—previously missing in the state-of-the-art—for the design of smart prosthetic socket solutions for transfemoral amputees.

## Conclusion

This study aimed at investigating volume fluctuations in the transfemoral amputee population due to the prosthesis doffing and physical activity, at different testing times (i.e., morning and afternoon). The results of these tests demonstrated a significant increase in volume following both prosthesis removal and 15-min of walking. In addition, the interaction of the three factors—prosthesis removal, physical activity and testing time—was found statistically significant. A two terms decay exponential function showed excellent fitting with the mean data of the post-doffing volume fluctuations over a 60 min period, demonstrating the highest change rate in the initial 10 min after the socket removal and an average stabilization time of 30 min. Considering volume fluctuations of each subject, values measured during this study were found within − 4.2 to + 2.7% (mean $\pm$ std: + 0.4% $\pm$ 1.2%) due to the prosthesis removal and − 1.4 to + 3.2% (mean $\pm$ std: + 0.5% $\pm 1.0$%) due to physical activity, with maximum volume reductions measured in subject 12, which is the only amputee with a prosthetic suspension system not based on vacuum.

The reported results could be exploited, in the future, for the design of smart prosthetic sockets able to compensate the limb volume fluctuations over time, thus to maximize stability and comfort.

## Supplementary Information

Supplementary Video 1.

Supplementary Information 1.
