# Supplementary material for: Residual limb volume fluctuations in transfemoral amputees
Source: Sci Rep. 2021 Jun 10;11:12273. doi: 10.1038/s41598-021-91647-9 (PMC8192500; doi:10.1038/s41598-021-91647-9)
Supplement: Supplementary file 2 — Supplementary Information 1. [file 41598_2021_91647_MOESM2_ESM.pdf]

Residual limb volume fluctuations in transfemoral amputees

Linda Paternò[1,2]*, Michele Ibrahimi[1,2], Elisa Rosini[1,2], Giuseppe Menfi[1,2], Vito Monaco[1,2,3], Emanuele Gruppioni[4], Leonardo Ricotti[1,2], and Arianna Menciassi[1,2]

[1] the BioRobotics Institute, Scuola Superiore Sant'Anna, Pisa, Italy.
[2] Department of Excellence in Robotics& AI, Scuola Superiore Sant'Anna, Pisa, Italy.
[3] IRCCS Fondazione Don Carlo Gnocchi, 20148 Milan, Italy
[4] INAIL Centro Protesi, Bologna, Italy.
*Corresponding e-mail: linda.paterno@santannapisa.it

Table S1. Previous clinical studies on stabilized lower residual limb volume changes. n°: number of enrolled subjects; TT: transtibial; TF: transfemoral; KD: knee disarticulation; VAS: Vacuum Assisted Suspension; A: Anterior; P: Posterior. The analysis of the literature was based on full papers only (*i.e.*, conference abstracts were excluded).

**Absolute volume changes:**	**Method**	**Results***	**n°**
Due to prosthesis doffing [1]	Custom scanning system	Max: 3.7%	1 TT
Comparing suction and vacuum at 23 inHg [2]	Water displacement with casts	Vacuum: -1.6% ÷ 8.5% (mean: 3.7%)<br>Suction: -11.3% ÷ -1.7% (mean: -6.5%)	10 TT
Comparing 4% under-sized, neutral and 4% over-sized [3]	Water displacement with casts	Under-sized: -1.79%<br>Neutral: +7.03%<br>Over-sized: + 12.85%	7 TT
Mean daily maximum [4]	Measuring liquid volume changes in a reservoir connected to the socket	Mean max: 2.7%	1 TF
In the long term (1 year or more) [5]	Water displacement	Range: +5% - +21%<br>Mean: 12%	7 TF, 1 KD, 6 TT
Due to prosthesis doffing and in 2 weeks [6]	Custom scanning system	Doffing: 2.4% ÷ 10.9% (mean 6.0%)<br>2 weeks: –2.0% ÷ +12.6% (mean 0.6%)	6 TT
Within a day and in the long term [7]	Custom scanning system	Day: –2.4% ÷ +2.2%<br>Long term: -4% ÷ 5%	8 TT
Due to prosthesis doffing and muscle contraction [8]	Custom scanning system	Post doffing: 3.3%<br>Muscle contraction < 1 %	1 TT
Due to daily activities [9]	VITUS/Smart 3D scanner	Range: 0.5% ÷ 7.5%	2 TF<br>1 TT
Comparing suction, vacuum at 10 inHg and 15 inHg [10]	OMEGA 3D scanner	Suction: 1.5% ÷ 4.9%<br>Vacuum 15 inHg: -0.5% ÷ -0.8%<br>Vacuum 10 inHg: 0.25% ÷ 0.8%	1 TT
Due to prosthesis doffing before and after activity, comparing VAS and pin suspension [11]	Custom scanning system	Pin: post activity: +4.5%<br>VAS: pre activity: 4.1%<br>VAS: post activity: 6.3%	5 TT

*volume changes are expressed as percentage changes with respect to the reference volume.

**Relative volume changes:**	**Method**	**Results****	**n°**
During sitting, standing, and walking, in the morning and in the afternoon [12]	Bioimpedance analyzer	Within-session: -8.5%/h - +5.9%/h (median: -2.2%/h)<br>Between-session -2.7 - +0.9%/h (median: -1.0 %/h)	12 TT
Comparing elevated and not elevated vacuum suspension [13]	Bioimpedance analyzer	Suction: -0.7% ÷ 4.3%<br>Pin: -2.1% ÷ 4.3%<br>VAS: -1.6% ÷ 3.4%	7 TT
Due to prosthesis doffing and physical activity [14]	Bioimpedance analyzer	Sit: 1.8% ± 1.4%<br>Liner: 1.3% ± 0.9%<br>Walk: 2.8% ± 2.0%	22 TT
Due to socks addition and removal within the socket [15]	Bioimpedance analyzer	Addition: -4.0% ÷ 0.8% (mean ± std: -0.9% ± 1.3%)<br>Removal: -1.2% ÷ 2.8% (mean ± std: 0.5% ± 0.8%)	28 TT

During resting, standing, walking [16]	Bioimpedance analyzer	Resting: -1.9% ÷ 5.7% (mean ± std: 1.0% ± 2.2%)<br>Standing:-5.4% ÷ -0.7% (mean ± std: -2.6% ± 1.1%)<br>Walking: -4.9% ÷ 5.7% (mean ± std: 1.0% ± 2.5%)	24 TT
With liquid-in and liquid-out bladders in the liner [17]	Bioimpedance analyzer	A: 0.4% ± 0.5%<br>P: 0.2% ± 0.7%	8 TT
When (1) prosthesis and liner donned; (2) prosthesis doffed and liner donned; (3) prosthesis and liner doffed [18]	Bioimpedance analyzer	(1) A:-2.1% ± 1.2%; P: -2.2% ± 1.1%<br>(2) A: 6.3% ± 3.5%; P: 6.1% ± 4.7%<br>(3) A: 4.9% ± 2.9%; P: 5.5% ± 3.0%	16 TT
During morning and afternoon [19]	Bioimpedance analyzer	Resting: −7.6% ÷ 10.3%<br>Standing: −15.6% ÷ 1.6%<br>Walking: −6.1% ÷ 11.4%	29 TT
In diabetic and nondiabetic amputees [20]	Bioimpedance analyzer	Diabetic: –6.9% ÷ 8.5%<br>Nondiabetic: -3.6% ÷ 4%	4 TT
Due to activity [21]	Bioimpedance analyzer	Low activity: –21.02 ÷ 1.01<br>High activity: –15.97 ÷ 7.51	13 TT
During resting, standing, walking [22]	Bioimpedance analyzer	Standing: -2.0% to -0.9%<br>Walking: -3.5% to +0.9%	4 TT
Comparing elevated vacuum and suction suspension [23]	Bioimpedance analyzer	VAS – P: -3.25%/h - -0.03%/h (mean ± std: -1.15%/h ± 0.92%/h)<br>VAS - A: -2.52%/h - -2.72%/h (mean ± std: -1.07%/h ± 0.86%/h)<br>Suction – P: -3.31%/h - +0.25%/h (mean ± std: -1.27%/h ± 1.02%/h)<br>Suction - A: -2.72%/h - +0.08%/h (mean ± std: -1.20 %/h ± 0.80%/h)	12 TT
Within motor-driven 3-panel adjustable sockets [24]	Bioimpedance analyzer	Enlargement: −0.2% - +4.5% (median: +1.5%)<br>Reduction: −8.0% - -0.7% (median: −1.7%)	10 TT

** extracellular fluid volume fluctuations in conductive tissue

Table S2. Residual limb volumes [dm^3] for each subject (S), measured during the 1st test session over the 60-minute period after the prosthesis doffing.

S	*time*						
	**0 min**	**10 min**	**20 min**	**30 min**	**40 min**	**50 min**	**60 min**
**S1**	2.15	2.16	2.17	2.17	2.16		
**S2**	2.08	2.15	2.15	2.16	2.15	2.15	2.15
**S3**	2.60	2.63	2.64	2.63	2.63	2.63	2.64
**S4**	3.36	3.37	3.37	3.41	3.39		
**S5**	3.60	3.61	3.61	3.63	3.64	3.64	3.64
**S6**	2.37	2.41	2.42	2.48	2.43	2.46	2.42
**S7**	1.95	1.97	1.99	2.02	2.02	2.01	1.99
**S8**	3.53	3.54	3.60	3.58	3.59	3.59	3.61
**S9**	2.96	2.98	2.99	2.99	2.99	2.97	3.00
**S10**	3.20	3.22	3.32	3.27	3.34	3.36	3.39
**S11**	2.83	2.85	2.84	2.85	2.86	2.86	2.87
**S12**	1.02	1.06	1.06	1.03	1.04	1.05	1.04
**S13**	3.15	3.28	3.19	3.21	3.17	3.25	3.16
**S14**	3.22	3.22	3.22	3.23	3.23	3.24	3.24
**S15**	2.33	2.34	2.35	2.34	2.35	2.34	2.39
**S16**	1.87	1.88	1.87	1.88	1.87	1.89	1.87
**S17**	2.65	2.68	2.69	2.65	2.72	2.67	2.69
**S18**	2.07	2.09	2.08	2.10	2.10	2.10	2.10
**S19**	1.85	1.85	1.85	1.85	1.87	1.86	1.86
**S20**	1.57	1.58	1.57	1.62	1.58	1.58	1.60
**S21**	3.25	3.40	3.32	3.36	3.40	3.34	3.37
**S22**	1.41	1.41	1.44	1.45	1.45	1.44	1.42
**S23**	4.42	4.43	4.43	4.46	4.48	4.47	4.59
**S24**	3.33	3.37	3.33	3.36	3.39	3.33	3.38

Table S3. Residual limb volumes[dm^3] for each subject (S), measured immediately after the prosthesis doffing and after stabilization, both before and after physical activity, and both in the morning and in the afternoon. Each volume value is the mean over the three different test days (*i.e.*, 2nd, 3rd, 4th test sessions).

S	*Morning*				*Afternoon*			
	Before activity		*After activity*		*Before activity*		*After activity*	
	Immediately after doffing	*After stabilization*	*Immediately after doffing*	*After stabilization*	*Immediately after doffing*	*After stabilization*	*Immediately after doffing*	*After stabilization*
**S1**	2.47	2.48	2.49	2.50	2.46	2.48	2.47	2.50
**S2**	2.04	2.04	2.04	2.04	2.02	2.03	2.02	2.03
**S3**	2.86	2.91	2.87	2.90	2.84	2.88	2.84	2.85
**S4**	2.87	2.88	2.91	2.96	2.86	2.90	2.92	2.93
**S5**	3.99	3.96	3.99	3.99	3.90	3.93	3.97	3.97
**S6**	2.53	2.54	2.54	2.57	2.52	2.54	2.54	2.58
**S7**	1.93	1.95	1.97	1.94	1.97	1.96	1.96	1.95
**S8**	2.86	2.84	2.86	2.88	2.82	2.81	2.85	2.83
**S9**	3.00	3.02	3.00	2.99	2.99	3.01	3.00	2.99
**S10**	3.23	3.24	3.24	3.28	3.30	3.33	3.25	3.29
**S11**	3.28	3.31	3.34	3.32	3.26	3.29	3.29	3.28
**S12**	0.68	0.66	0.69	0.66	0.69	0.67	0.70	0.69
**S13**	2.71	2.69	2.70	2.71	2.68	2.69	2.68	2.70
**S14**	2.52	2.56	2.54	2.61	2.52	2.58	2.54	2.57
**S15**	2.09	2.12	2.13	2.12	2.07	2.08	2.12	2.11
**S16**	1.61	1.62	1.62	1.62	1.61	1.61	1.62	1.63
**S17**	2.66	2.66	2.63	2.65	2.64	2.65	2.68	2.68
**S18**	1.70	1.71	1.68	1.72	1.67	1.71	1.69	1.69
**S19**	1.85	1.84	1.85	1.83	1.86	1.85	1.85	1.85
**S20**	1.65	1.65	1.67	1.68	1.68	1.70	1.68	1.70
**S21**	3.35	3.37	3.31	3.36	3.27	3.33	3.27	3.32
**S22**	1.41	1.39	1.42	1.42	1.38	1.41	1.41	1.39
**S23**	4.43	4.44	4.48	4.48	4.43	4.54	4.51	4.49

References

1. Sanders, J. E., Greve, J. M., Clinton, C. & Hafner, B. J. Changes in interface pressure and stump shape over time: Preliminary results from a trans-tibial amputee subject. *Prosthet. Orthot. Int.* **24**, 163–168 (2000).
2. Board, W. J., Street, G. M. & Caspers, C. A comparison of trans-tibial amputee suction and vacuum socket conditions. *Prosthet. Orthot. Int.* **25**, 202–209 (2001).
3. Goswami, J., Lynn, R., Street, G. & Harlander, M. Walking in a vacuum assisted socket shifts the stump fluid balance. *Prosthet. Orthot. Int* **27**, 107–113 (2003).
4. Greeenwald, R. M., Dean, R. C. & Board, W. J. Volume management: smart variable geometry socket (SVGS) technology for lower-limb prostheses. *Prosthet. Orthot. Int* **15**, 107–112 (2003).
5. Fernie, G. R. & Holliday, P. J. Volume fluctuations in the residual limbs of lower limb amputees. *Arch. Phys. Med. Rehabil.* **63**, 162–165 (1982).
6. Zachariah, S. G., Saxena, R., Fergason, J. R. & Sanders, J. E. Shape and volume change in the transtibial residuum over the short term: preliminary investigation of six subjects. *J. Rehabil. Res. Dev.* **41**, 683–94 (2004).
7. Sanders, J. E., Zachariah, S. G., Jacobsen, A. K. & Fergason, J. R. Changes in interface pressures and shear stresses over time on trans-tibial amputee subjects ambulating with prosthetic limbs: Comparison of diurnal and six-month differences. *J. Biomech.* **38**, 1566–1573 (2005).
8. Solav, D., Moerman, K. M., Jaeger, A. M. & Herr, H. M. A Framework for Measuring the Time-Varying Shape and Full-Field Deformation of Residual Limbs Using 3-D Digital Image Correlation. *IEEE Trans. Biomed. Eng.* **66**, 2740–2752 (2019).
9. Staker, M., Ryan, K. & LaBat, K. Medicine and Design Investigate Residual Limb Volume Fluctuations: Three Case Studies. *Australas. Med. J.* 156–161 (2008). doi:10.4066/AMJ.2009.92
10. Gerschutz, M. J., Denune, J. A., Colvin, J. M. & Schober, G. Elevated Vacuum Suspension Influence on Lower Limb Amputee's Residual Limb Volume at Different Vacuum Pressure Settings. *JPO J. Prosthetics Orthot.* **22**, 252–256 (2010).
11. Klute, G. K. *et al.* Vacuum-assisted socket suspension compared with pin suspension for lower extremity amputees: Effect on fit, activity, and limb volume. *Arch. Phys. Med. Rehabil.* **92**, 1570–1575 (2011).
12. Sanders, J. E. *et al.* Preliminary investigation of residual-limb fluid volume changes within one day. *J. Rehabil. Res. Dev.* **49**, 1467–1478 (2012).

13. Sanders, J. E., Harrison, D. S., Myers, T. R. & Allyn, K. J. Effects of elevated vacuum on in-socket residual limb fluid volume: Case study results using bioimpedance analysis. *J. Rehabil. Res. Dev.* **48**, 1231–1248 (2011).
14. Sanders, J. E. *et al.* Post-doffing residual limb fluid volume change in people with trans-tibial amputation. *Prosthet. Orthot. Int.* **36**, 443–449 (2012).
15. Sanders, J. E. *et al.* How do sock ply changes affect residual-limb fluid volume in people with transtibial amputation? *J. Rehabil. Res. Dev.* **49**, 241–256 (2012).
16. Sanders, J. E., Cagle, J. C., Allyn, K. J., Harrison, D. S. & Ciol, M. A. How do walking, standing, and resting influence transtibial amputee residual limb fluid volume? *J. Rehabil. Res. Dev.* **51**, 201–212 (2014).
17. Sanders, J. E. *et al.* Preliminary evaluation of a novel bladder-liner for facilitating residual-limb fluid volume recovery without doffing. *J. Rehabil. Res. Dev.* **53**, 1107–1120 (2016).
18. Sanders, J. E. *et al.* Does temporary socket removal affect residual limb fluid volume of trans-tibial amputees? *Prosthet. Orthot. Int.* **40**, 320–328 (2016).
19. Sanders, J. E. *et al.* Residual limb fluid volume change and volume accommodation: Relationships to activity and self-report outcomes in people with trans-tibial amputation. *Prosthet. Orthot. Int.* **42**, 415–427 (2018).
20. Sanders, J. E., Rogers, E. L. & Abrahamson, D. C. Assessment of residual-limb volume change using bioimpedence. *J. Rehabil. Res. Dev.* **44**, 525–35 (2007).
21. Youngblood, R. T. *et al.* Effects of activity intensity, time, and intermittent doffing on daily limb fluid volume change in people with transtibial amputation. *Prosthet. Orthot. Int.* **43**, 28–38 (2019).
22. Sanders, J. E., Harrison, D. S., Allyn, K. J. & Myers, T. R. Clinical Utility of In-Socket Residual Limb Volume Change Measurement: Case Study Results. *Prosthet. Orthot. Int.* **33**, 378–390 (2009).
23. Youngblood, R. T. *et al.* Effectiveness of elevated vacuum and suction prosthetic suspension systems in managing daily residual limb fluid volume change in people with transtibial amputation Original Research Report. *Prosthet. Orthot. Int.* **44**, 155–163 (2020).
24. McLean, J. B. *et al.* Socket size adjustments in people with transtibial amputation: Effects on residual limb fluid volume and limb-socket distance. *Clin. Biomech.* **63**, 161–171 (2019).
